# Comparative study between antegrade flexible ureteroscopy and reterograde intrarenal surgery in the management of impacted upper ureteric stones 1.5 cm or larger

**DOI:** 10.1007/s00345-023-04672-w

**Published:** 2023-11-03

**Authors:** Ahmed Mohey, Ahmed A. Abdelfattah, Ahmed E. Mohammed, Abdelmoniem Marzouk, Amr S. El-Dakhakhny

**Affiliations:** Department of Urology, Benha University Hospital, Faculty of Medicine, Benha University, Farid Nada Street, Benha, Egypt

**Keywords:** Antegrade ureteroscopy, Reterograde intrarenal surgery, Upper ureteric stones

## Abstract

**Objective:**

To prospectively investigate the safety and efficacy of antegrade flexible ureteroscopy (FURS) with the following criteria (supine, ultrasonic guided puncture through lower calyx with 14 fr tract, tubeless) versus retrograde intrarenal surgery (RIRS) in the management of large impacted upper ureteric stones ≥ 1.5 cm.

**Patients and methods:**

This study recruited 61 patients with single large impacted upper ureteric stone of ≥ 1.5 cm. The patients were randomly allocated to two groups. Group A, included 31 patients who treated by antegrade FURS, all patients were put in supine modified galadako Valdivia position and the renal access is reached by ultrasonic guided puncture through the lower calyx with dilatation upto 14 fr to insert ureteric access sheath and all cases were tubless with JJ stent insertion. Group B, included 30 patients who were treated by RIRS with JJ stent insertion. Stone fragmentation was done by holmium laser in both group.

**Results:**

Group A was significantly associated with higher proportion of SFR (90.3%) compared to Group B (70%) (p = 0.046). Group B was significantly associated with shorter operative time and fluoroscopy time in comparison with Group A (p < 0.001). No significant differences were found between studied groups regarding bleeding (p = 0.238). Urosepsis showed significantly higher proportion associated with retrograde approach when compared to antegrade approach (p = 0.024).

**Conclusion:**

This study showed that antegrade FURS is safe and more effective than RIRS for the management of large impacted upper ureteric stones ≥ 1.5 cm.

## Introduction

Impacted ureteral stones have different definitions as stones which not moved for two months or that not bypassable with contrast medium or with a guidewire [1, 2]. The management of patients with impacted large upper ureteric stone remains challenging [3, 4]. URS (semirigid or flexible) considered the most popular procedure for the management of upper third ureteric stone associated with the stone free rate (SFR) 89–100% with laser lithotripsy [5, 6]. However, the retrograde fashion of URS carries the risk of difficulty due to stone impaction on ureteral wall as a result of oedema, making the field of vision narrows increasing the possibility of complications, also the narrow caliber of the distal ureter makes the advance of URS more risky [7, 8]. Antegrade access through URS to upper ureteric stones is an option to manage them with the advantage of reaching the stone from dilated system, no risk of stone escape and better field of vision, however, this antegrade mode bears the drawbacks of being invasive due to renal puncture, more exposure to radiation, more operative time and more hospital stay [9]. We have done puncture in the lower calyx and dilatation up to 14 fr with the hypothesis that this would minimize the invasive nature of this procedure. In this study, we prospectively investigated the safety and efficacy of antegrade flexible ureteroscopy (FURS) versus retrograde intrarenal surgery (RIRS) in the management of impacted large upper ureteric stone ≥ 1.5 cm.

## Patients and methods

### Study design

This prospective randomized study was conducted at Benha university hospital, urology department in the period from February 2022 to February 2023. All patients applied written informed consent and our local ethical committee approval was obtained (MD.9.2.2022). The inclusion criteria involved patients aged ≥ 18 years with single large impacted upper ureteric stones (located just below PUJ and above the lower limit of the fourth lumber vertebra) ≥ 1.5 cm in maximum dimension. Patients with kidney stones, obstruction distal to the stone, active UTI and pregnant females were excluded.

Patients were randomly divided into two groups using a closed-envelopes method.

Group A: included 31 patients who treated by antegrade FURS procedure and holmium laser fragmentation.

Group B: included 30 patients who treated by retrograde FURS procedure and holmium laser fragmentation.

All patients subjected to complete history taking, physical examinations. Urine analysis, urine culture, serum creatinine, complete blood count, hepatitis marker and coagulation profile were assessed as preoperative laboratory investigations. Patients with positive urine culture received the specific antibiotic up to the urine culture registered negative. while patients with negative urine culture received a single dose of first generation cephalosporin for operation. Radiological investigations for all patients were done, including pelvic-abdominal ultrasonography (us), plain radiograph of the kidneys, ureters and bladder (KUB) and non-contrast CT to determine stone criteria.

### Operative technique

All procedures carried out by two consultants and experienced endourologist.

### Group A: Antegrade flexible ureteroscopy procedure (Antegrade FURS)

**Fig. 1:**
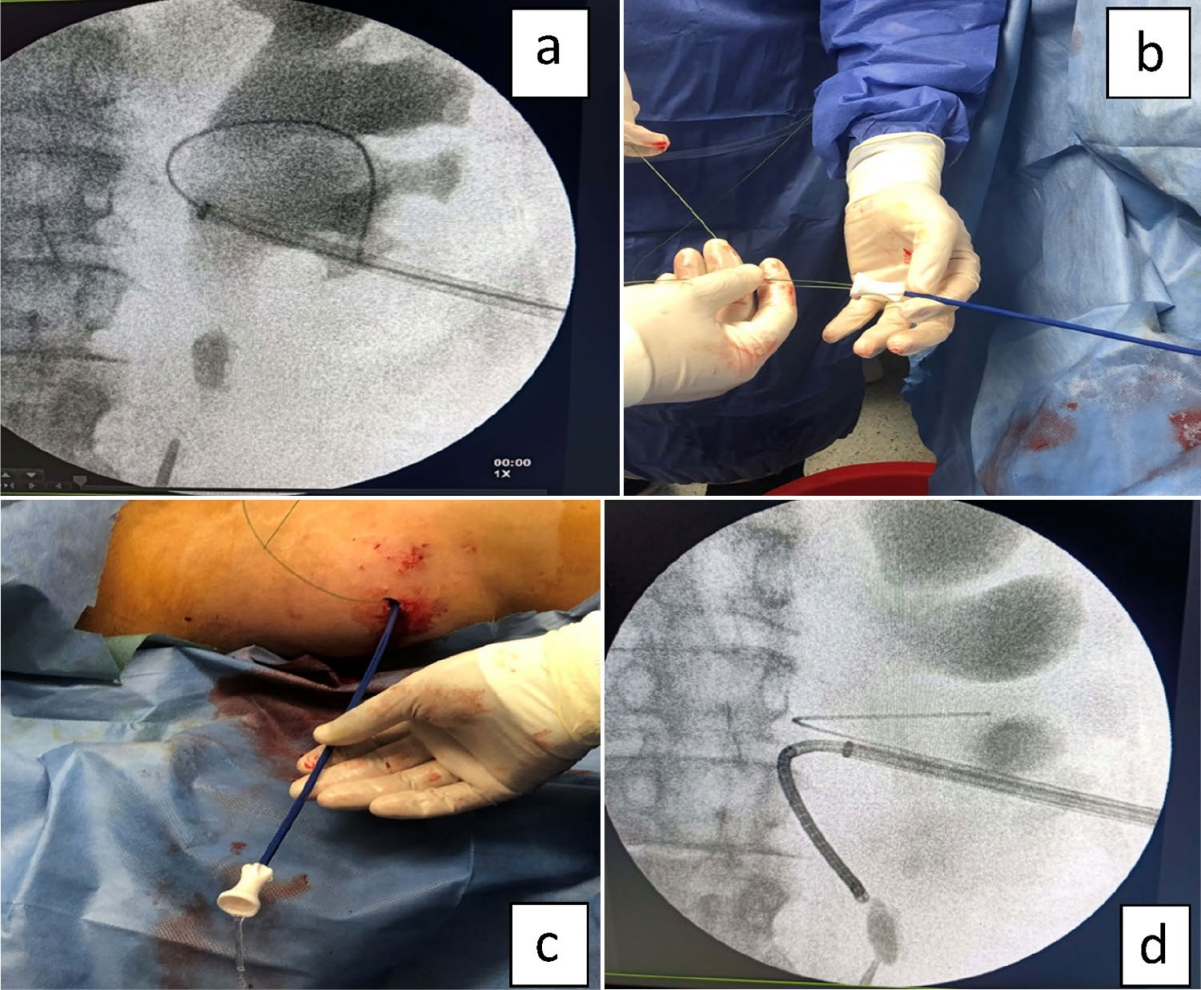
**a** Introduction of ureteral access sheath over the guide wire. **b** Another guide wire insertion through access sheath. **c** Safety guide wire and access sheath inside collecting system. **d** Flexible URS reaching the stone

Under spinal anesthesia, patients were moved to Supine Modified Galadako Valdiva Position as this position permitted simultaneous antegrade and retrograde access so the patients were drapped once and repositioning was not required. Cystoscopy was performed and 6 fr ureteric catheter was inserted into targeted ureter just below the stone, as passage of ureteric cathter in ease considered as a marker of no significant distal obstruction and also, act as a landmark when appear in the field that the stone is completely fragmented, then cystoscope removed and 16 fr bladder catheter was inserted which fixed to the distal end of ureteric catheter. While the patients in the same position and under the ultrasound guidance the collecting system was punctured through the lower calyx. After confirming the safe placement of the needle into the collecting system by free urine flow, the contrast agent was injected through the needle to opacify the collecting system under fluoroscopy. After which a guidewire (0.038) was inserted into the collecting system through the needle and skin incision was made then the needle removed. Dilatation of the tract up to 14 fr was done using amplatz dilators followed by introduction of ureteral access sheath (UAS) (Navigator 13/11 F, 46 cm: Boston Scientific) over the guide wire (Fig. 1a). Through the access sheath, we inserted another guidewire into the collecting system (Fig. 1b). The access sheath was then removed, one of the two guidewires was used as safety wire while the second was used to introduce the access sheath again into the collecting system(Fig. 1c) then removed to introduce the flexible URS (9.5 Fr The LithoVue™ System Boston Scientific) through access sheath until reach the stone (Fig. 1d). The stone was fragmented using the holmium laser (Lumenis® Pulse™30H) with energy 0.8–1 J per pulse and repetition rate 6–10 HZ. At the end of the procedure, the JJ stent was inserted in antegrade manner and the access sheath removed without inserting a nephrostomy tube.

### Group B: Reterograde flexible ureteroscopy procedure (Retrograde FURS)

Under spinal anesthesia, patients were put in the dorsal lithotomy position. Cystoscopy was performed and two guidewire were inserted in the targeted ureter. One of them was used as safety wire while the other was used for advancing the flexible URS upto the stone. Before the reterograde FURS maneuver, ureteral dilatation was done by balloon dilator if needed. Flexible URS was performed with a 9.5 Fr The LithoVue™ System-Boston Scientific. The stones were fragmented with the same laser device and settings as in group A. At the end of the procedure JJ stent was inserted in a reterograde manner.

In both groups, the irrigation flow depended on gravity- based irrigation using saline and fragments were left for spontaneous passage after fragmentation into very tiny fragments.

Both groups were compared as regard preoperative data (Age, Sex, stone characteristics), intra-operative data (Operative Time, Lithotripy time, Fluroscopy time, complications), and postoperative outcomes (stone free rate, complications, need for auxiliary procedure).

Operative time was defined in both groups as the time from cystoscopy until JJ stent insertion. Two weeks after the procedure in both groups, all patients undergone non contrast CT before JJ stent removal to assess SFR which was defined as absence of residual fragment ˃ 3 mm.

The primary endpoint of the present study was the SFR at 2 weeks. The secondary endpoint included intraoperative data (operative time, time for lithotripsy and fluoroscopy) and postoperative complications.

### Statistical analysis

The Statistical package for Social Science (IBM Corp. Released 2017. IBM SPSS Statistics for Windows, Version 25.0. Armonk, NY: IBM Corp.) was used for data management and analysis. Descriptive statistics were presented as Mean, Standard deviation (± SD) or numbers and percentages. Chi-Square test was used to assess the relationship between two qualitative variables. Student T Test was used to detect the statistical significance of the difference of the parametric variable between two study group means. Fisher’s exact test was used to estimate the relationship between two qualitative variables. The P value is considered significant if < 0.05.

## Results

The Patients and stone criteria are described in Table 1. The age of patients was 45.19 ± 7.58 years in group A and 44.93 ± 6.87 years in group B. In group A, male patients represented 51.6% and female was 48.4%, while in group B male patients were 56.7% and female represented 43.3%. There was no significant difference between the two groups as regard patients sex and age. The stone size was 17.61 ± 2.19 mm in group A while 17.40 ± 2.21 mm in group B. As regards stone side, size, density there was no significant difference between both groups. Operative and post-operative data are mentioned in Table 2. Group B was significantly associated with shorter operative time 64.40 ± 7.16 min and Fluoroscopy time 112.33 ± 20.29 s in comparison with Group A 93.55 ± 7.58 min and 254.84 ± 46.82 s (p < 0.001). As regards lithotripsy time in group A (35.68 ± 2.71 min) and group B (35.20 ± 2.87 min) no significant difference detected (p = 0.507). Group A was significantly associated with higher proportion of SFR (90.3%) compared to Group B (70%) (p = 0.046). Patients who treated with retrograde approach, required significantly higher proportion of auxiliary treatment as 30% had ESWL, while only 9.3% of those subjected to antegrade approach required ESWL (p = 0.046). Urosepsis showed significantly higher proportion associated with retrograde approach when compared to antegrade approach (16.7% versus 0%, p = 0.024), and all were treated by empirical antibiotic. No significant differences were found between studied groups regarding bleeding (p = 0.238) however, three cases of bleeding (less than 150 cc) in Group A were managed conservatively and no need for blood transfusion. No significant difference was found between Group A and Group B as regard VAS score (6.5% versus 0%, p > 0.05).

**Table 1:** Patients and stone characters

Variable	Group A Antegrade FURS (N = 31)	Group B RIRS (N = 30)	Test	p value
Age, years, mean (SD)	45.19 ± 7.58	44.93 ± 6.87	t = 0.140	0.889
Sex, n (%)				
Male	16 (51.6)	17 (56.7)	X2 = 0.157	0.692
Female	15 (48.4)	13 (43.3)		
Stone site, n(%)				
L2-3	5 (16.1)	5 (16.7)	X2 = 0.178	0.981
L3	7 (22.6)	6 (20.0)		
L3-4	13 (41.9)	12 (40)		
L4	6 (19.4)	7 (23.3)		
Stone size, mm, mean (SD) (Range)	17.61 ± (2.19) (15–25)	17.40 ± 2.21 (15–25)	t = 0.379	0.706
Stone density, HU, mean (SD)	980.9 ± 208.2	989.5 ± 188.3	t = 0.168	0.867
Stone side, n (%)				
Right	14 (45.2)	13 (43.3)	X2 = 0.021	0.886
Left	17 (54.8)	17 (56.7)

**Table 2:** Operative and post-operative data

Variable	Group A Antegrade FURS (N = 31)	Group B RIRS (N = 30)	Test	*p* value
Operative time, min, mean (SD)	93.55 ± 7.58	64.40 ± 7.16	t = 15.433	< 0.001
Fluoroscopy time, s, mean (SD)	254.84 ± 46.82	112.33 ± 20.29	t = 15.508	< 0.001
Lithotripsy time, min, mean (SD)	35.68 ± 2.71	35.20 ± 2.87	t = 0.668	0.507
Complications n (%)				
Bleeding (< 150 cc)	3 (9.7)	0 (0)	X2 = 3.053	0.238
Sepsis	0 (0)	5 (16.7)	X2 = 5.628	0.024
Haematuria	3(9.7)	2(6.7)	X2 = 0.184	1.000
Stone free rate, n/N (%)	28/31(90.3)	21/30 (70.0)	X2 = 3.985	0.046
Auxiliary treatment (ESWL), n/N (%)	3/31 (9.7)	9/30 (30)	X2 = 3.985	0.046
VAS Score, n/N (%)				
1–3	29/31 (93.5)	30/30 (100)		
4–6	2/31 (6.5)	0/30 (0)	X2 = 2.001	0.492

## Discussion

RIRS represents the most common approach for skilled surgeons and for patients as it can be done through the nature orifices and seems to be minimally invasive. However, RIRS has some drawbacks, especially for the management of large impacted upper ureteric stones such as low success rates, need multiple sessions, more complications as a result of a narrow field of vision due to the narrow space around the stone and risk of urosepsis particularly with prolonged operative time [10, 11]. Antegrade URS is an effective and safe alternative approach to RIRS in the management of impacted large upper ureteric stones with the advantage of higher SFR, wide field of vision due to dilated upper ureter however, antegrade URS carry some risks of bleeding due to renal puncture especially middle and upper calyx puncture, more radiation exposure and more operative time [9].

We prospectively designed the present study trying to minimize the drawbacks of antegrade approach especially the bleeding due to its invasive nature by doing a puncture through lower calyx and tract dilatation to 14 fr.

So, we think that to the best of our knowledge this is the first study done in prospective manner and totally supine, ultrasonic guided puncture through the lower calyx with 14 fr tract, tubeless for antegrade FURS in the management of impacted large upper ureteric stones ≥ 1.5 cm.

In the present series, SFR was higher in the antegrade URS group than in RIRS group after 2 weeks 90.3% vs. 70% p = 0.046. This significant difference may be due to better field of vision as the procedure done through dilated system and the very rare possibility of stone movement. This was close to the rates documented by various studies clarifying the upper hand of antegrade approach such as Liu et al. [12] and Elgebaly et al. [13] who reported higher stone free rate in antegrade group in comparsion with reterograde group 97.7% vs 82.2% and 83.3 vs 60% respectively.

In the present study, the operative time was shorter in RIRS group than antegrade FURS group 64.40 (± 7.16) vs 93.55 (± 7.58) min p < 0.001. This significant difference was due to time taken for renal puncture, dilatation of the tract and manipulation for direction of flexible URS until reaching the stone into ureter.This results was congruent with many published studies which reported longer operative time in the antegrade procedure. Elgebaly et al. [13] conducted a prospective study on 60 patients comparing between antegrade mini-percutaneous URS in prone position through middle and upper calyx and reterograde URS either by semirigid or flexible URS in the management of upper ureteric stone ˃ 1 cm. The study reported that the operative time was significantly shorter in the retrograde URS group than antegrade miniperc URS group 64.7 (± 17.7) vs. 112 (± 15.3) min. Also Li et al. [14] mentioned that operative time was longer in a percutaneous nephrolithotomy group than for retrograde URS group 108.76 (± 19.36) vs. 63.56 (± 16.38) min respectively (p < 0.05).

In the present study, bleeding less than 150 ml occurred in three cases in antegrade FURS group and all cases managed conservatively and no need for blood transfusion while no cases in RIRS group. As the bleeding is considered the most nightmares of this antegrade URS maneuver due to its invasive nature, but we think that our technique introduce almost safe way for this procedure as we done puncture through lower calyx which carry less risk of bleeding than middle and upper calyceal puncture using ultrasonic guidance with dilatation to only 14 fr. Urosepsis occurred in five cases of RIRS group while no cases in antegrade FURS group confirming that urosepsis is a technique related complication due to high pelvicalyceal pressure as we excluded patient related factor for urosepsis.

In literature, the reported overall complications in both groups are comparable. Elgebaly et al. [13] reported four cases of bleeding in antegrade URS procedure while there was no bleeding in retrograde group. Güler and Erbin [15] reported four cases who developed urosepsis in RIRS procedure and no patient in the antegrade URS procedure.

From our practice we noticed that the antegrade FURS approach is a promising procedure for the management of large impacted upper ureteric stones as minitract (14 Fr) through the lower calyx using ultrasonic guidance carry less risk of bleeding, easy manipulation and direction of flexible URS through the ureter with high SFR. We think that shorter UAS and scopes should be designed for this particular percutaneous procedure in the future. Also, we recommended more studies to compare different instrumentations such as ultraminiPERC sheath and UAS for antegrade approach.

The limitations which touched our study, including that the study done in a single center. We hope to see several randomized studies with more number of patients and longer follow up periods from other centers for further confirmation of the safety and efficacy of antegrade FURS in the management of impacted large upper ureteric stones ≥ 1.5 cm.

## Conclusion

Antegrade FURS for the management of large impacted upper ureteric stones ≥ 1.5 cm via totally supine position, ultrasonic guided through the lower calyx with minitract 14 fr and tubless being a safe and feasible procedure, with high SFR, no significant bleeding and less incidence of sepsis but with more operative time and radiation exposure. The high success rate and few rate of complications encountered, suggest that antegrade FURS will be a promising procedure for management of large impacted upper ureteric stones in this size group (≥ 1.5 cm).

## Data Availability

Data available upon reasonable request from the authors.
